# Mathematical bounds on Shannon entropy given the abundance of the *i*th most abundant taxon

**DOI:** 10.1007/s00285-023-01997-3

**Published:** 2023-10-26

**Authors:** Maike L. Morrison, Noah A. Rosenberg

**Affiliations:** Department of Biology, Stanford University, Stanford, CA 94305 USA

**Keywords:** Biodiversity, Diversity indices, Majorization, Shannon entropy, Species richness, 92D40, 94A17, 92D10

## Abstract

The measurement of diversity is a central component of studies in ecology and evolution, with broad uses spanning multiple biological scales. Studies of diversity conducted in population genetics and ecology make use of analogous concepts and even employ equivalent mathematical formulas. For the Shannon entropy statistic, recent developments in the mathematics of diversity in population genetics have produced mathematical constraints on the statistic in relation to the frequency of the most frequent allele. These results have characterized the ways in which standard measures depend on the highest-frequency class in a discrete probability distribution. Here, we extend mathematical constraints on the Shannon entropy in relation to entries in specific positions in a vector of species abundances, listed in decreasing order. We illustrate the new mathematical results using abundance data from examples involving coral reefs and sponge microbiomes. The new results update the understanding of the relationship of a standard measure to the abundance vectors from which it is calculated, potentially contributing to improved interpretation of numerical measurements of biodiversity.

**Supplementary Information:**

The online version contains supplementary material available at 10.1007/s00285-023-01997-3.

## Introduction

The quantitative measurement of features of biological diversity is central to ecology. Over decades of analysis, many statistics have been proposed as diversity measures, and their mathematical properties have been studied (Pielou 1975; Magurran 2004; Leinster 2021).

Among the enduring measures of diversity in ecology is the Shannon entropy, first borrowed in the 1950s from the formula’s origins with Shannon’s information theory (Shannon 1948), and variously known as the Shannon diversity, Shannon index, Shannon-Weaver index, or Shannon-Wiener index (Spellerberg and Fedor 2003; Rodríguez et al. 2016; Sherwin and Fornells 2019). For a frequency vector $p = (p_1, p_2, \ldots , p_n)$, the Shannon entropy is

1 $$\begin{aligned} H(p)=-\sum _{i=1}^np_i\log p_i = \sum _{i=1}^nh(p_i), \end{aligned}$$

where each $p_i$ is a non-negative quantity that, in biodiversity measurement, represents the relative abundance of species *i* in a community. The $p_i$ sum to 1 and $h(p_i) = -p_i\log p_i$. We use the base-*e* logarithm and adopt the convention of defining $-0\log 0 = 0$ (Leinster 2021, pp. 39-40).

Shannon entropy has a number of convenient mathematical properties as a diversity measure for the species in a community. Considering all possible frequency vectors, it reaches its minimum of 0 when the vector has only one non-zero entry with frequency 1. Its maximum of $\log n$ is reached when the distribution of probabilities across *n* categories is uniform; the upper bound therefore increases with the vector length *n* (Leinster 2021, pp. 41-42). In the language of biodiversity, Shannon entropy is large when a community contains many equally common species, and it is minimal when the community has only one species. The Shannon entropy can be linked to broader families of statistics, such as the Rényi entropies (Rényi 1961), for which it can be regarded as a limiting case, and the Hill numbers (Hill 1973; Jost 2006; Leinster and Cobbold 2012; Chao et al. 2014), for which its exponential $e^H$ is a special case.

With its long-standing role as a popular diversity statistic, Shannon entropy is ubiquitous in biodiversity studies (Pielou 1975; Magurran 2004; Sherwin and Fornells 2019; Cushman 2021). Hence, new mathematical results concerning its behavior have the potential to assist in understanding features of numerous ecological communities, both in ongoing studies and in previously reported analyses that have relied upon this index.

A general aspect of diversity measurement is that a diversity statistic computed from frequency vectors, each representing the relative abundances of species in a community, can reach similar values for quite different species relative abundances. Consider two communities with different values for the Shannon entropy. Is the difference driven by abundance differences in one or two dominant species, or by differences in many less common species? Consider also two communities that have similar Shannon entropy values and whose abundances are similar only for the few dominant species that have the strongest influence on the numerical value of the statistic. Is the similarity meaningful in light of abundance differences among the rarer species?

We seek to provide insight on such questions by exploring the mathematical constraints, or bounds, imposed on Shannon entropy by the *i*th-most abundant species. That is, if we fix the frequency of the *i*th-most abundant species in a community but leave other frequencies free to vary, what are the largest and smallest possible values of Shannon entropy?

Working with the case of $i=1$ in a population genetics context mathematically identical to that used in ecological diversity computations, Aw and Rosenberg (2018) noted that if the frequency $p_1$ of the largest value in a frequency vector is fixed, then Shannon entropy is bounded above both by $\log n$ and by a tighter bound, a certain function of $p_1$. Further, the value of $p_1$ produces a certain lower bound on Shannon entropy. Thus, with $p_1$ specified, Shannon entropy is constrained more tightly than the interval $[0, \log n]$. If the Shannon entropy is computed in a community that possesses a single dominant species, then the placement of the Shannon entropy with respect to this tighter interval conditional on the abundance of the dominant species is perhaps a more meaningful value than its placement with respect to $[0, \log n]$. To better inform comparisons of biodiversity measurement among communities, the bounds we provide on Shannon entropy in relation to the frequency of the *i*th-most abundant species clarify the dependence of Shannon entropy on the relative abundances of the various species—not only the most abundant one.

## Bounds on Shannon entropy: the most abundant species

Similar values of Shannon entropy can be generated by quite different species composition vectors. For example, consider two communities, each with ten species in total. Community A has two moderately common species and eight rare species: one species at abundance 0.5, another at abundance 0.492, and eight rare species at abundance 0.001 each. Community B is dominated by a single species at abundance 0.85, with the remaining nine species each having abundance $\frac{1}{60}$. These communities both have Shannon entropy $H \approx 0.75$ despite having quite different composition. One way to contextualize the Shannon entropies of these two communities is to look at them in light of the upper and lower bounds on Shannon entropy conditional on the abundance of the most abundant species and the total number of species. This approach takes into account differing most-abundant-species abundances, allowing a researcher to understand if the values of Shannon entropy are chiefly a byproduct of the abundance of a single dominant species.

Aw and Rosenberg (2018) established the bounds on Shannon entropy as a function of the greatest abundance (Corollary 3.16). Without loss of generality, we re-order the species relative abundance vector *p* such that $p_1\geqslant p_2 \geqslant \ldots \geqslant p_n$. The distribution of abundance across the entries of the vector is constrained by two requirements: the entries must sum to 1, and $p_i\geqslant p_j$ if $i < j$.

### Proposition 1

For a fixed value of the frequency $p_1$ of the most abundant species in a community with *n* species, the vector maximizing *H* is $p^*$, where

$$\begin{aligned} p^* = \left( p_1, \frac{1-p_1}{n-1}, \frac{1-p_1}{n-1}, \ldots , \frac{1-p_1}{n-1}\right) . \end{aligned}$$

The upper bound on *H* is

2 $$\begin{aligned} H(p^*)=H_{\max }(p_1,n) = h(p_1) + (n-1) \, h\Big (\frac{1-p_1}{n-1}\Big ). \end{aligned}$$

### Proposition 2

For a fixed value of the frequency $$p_1$$ of the most abundant species in a community with *n* species, the vector minimizing *H* is $$p^{**}$$, where

$$\begin{aligned} p^{**} = \Big (p_1, p_1, \ldots , p_1, 1-\Big (\Big \lceil \frac{1}{p_1} \Big \rceil -1 \Big ) p_1, 0, \ldots , 0 \Big ), \end{aligned}$$

with the first $\lceil 1/p_1\rceil -1$ entries equal to $p_1$. The lower bound on *H* is

3 $$\begin{aligned} H(p^{**})=H_{\min }(p_1,n) = \Big (\Big \lceil \frac{1}{p_1}\Big \rceil -1 \Big ) h(p_1)+ h\Big (1-\Big (\Big \lceil \frac{1}{p_1}\Big \rceil -1\Big ) p_1\Big ). \end{aligned}$$

In general, Shannon entropy is greatest when the distribution of species is as “even” as possible, reaching its maximum $\log n$ across all *n*-species abundance distributions if the *n* species each have abundance $\frac{1}{n}$. In Proposition 1, if $p_1$ is fixed, then Shannon entropy is maximized when the remaining abundance, $1-p_1$, is spread evenly across all $n-1$ remaining species.

On the other hand, Shannon entropy is smallest when the distribution of species is as “uneven” as possible. Across all *n*-species abundance distributions, this minimum is obtained if a single species has abundance 1 and all other species have abundance 0. In Proposition 2, for fixed $p_1$, Shannon entropy is minimized when the remaining abundance, $1-p_1$, is distributed across as few species as possible. If $p_1\geqslant \frac{1}{2}$, then this minimizing vector is simply $(p_1, 1-p_1, 0, \ldots , 0)$. If, instead, $p_1<\frac{1}{2}$, then the condition $p_1 \geqslant p_2 \geqslant \ldots \geqslant p_n$ requires that none of the subsequent vector entries exceed $p_1$. The largest abundance we can assign to any one species is $p_1$, and we repeat this assignment as many times as possible before assigning all remaining abundance to (at most) one last species.

**Fig. 1:**
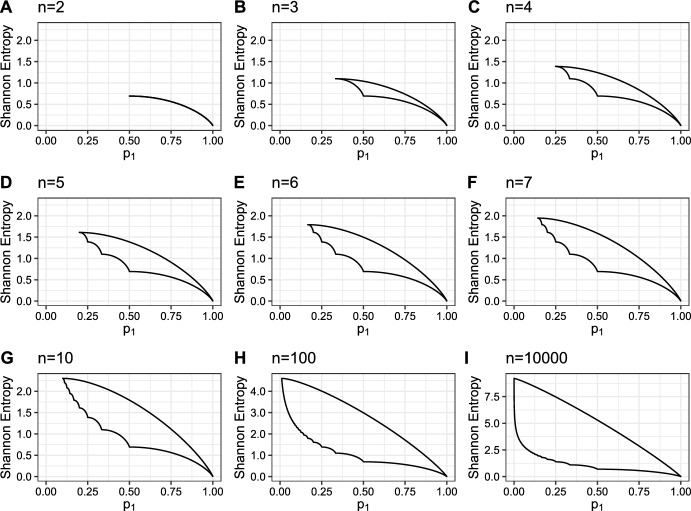
Upper and lower bounds on Shannon entropy as functions of the abundance of the most abundant species, $p_1$, for varying species richness, *n*. **A** $n=2$. **B** $n=3$. **C** $n=4$. **D** $n=5$. **E** $n=6$. **F** $n=7$. **G** $n=10$. **H** $n=100$. **I** $n=10{,}000$. For fixed *n*, *H* is maximized when $p=(\frac{1}{n}, \frac{1}{n}, \ldots , \frac{1}{n})$, with $H(\frac{1}{n},\frac{1}{n},\ldots ,\frac{1}{n})=\log n$. As the number of entries, *n*, increases, this upper bound, $\log n$, increases, so the range of the y-axis increases. The bounds are taken from Propositions 1 and 2. Note that panels H and I have y-axis scales that differ from those of the other panels

The upper and lower bounds on Shannon entropy are plotted as a function of $p_1$ for varying values of *n*, the number of species, in Fig. 1. Both bounds decrease with increasing $p_1$; the lower bound has a piecewise structure, reflecting the fact that fewer species have non-zero abundance as $p_1$ increases. Across panels with increasing *n*, the upper bound increases; the lower bound for a given $p_1$ remains the same, except that its domain grows with *n*.

To compare Communities A and B, we direct our attention to the vertical cross sections at $p_1 = 0.5$ and $p_1 = 0.85$ of Fig. 1G, which shows the bounds on Shannon entropy conditional on $p_1$ for communities with $n=10$ species. The large space between the upper and lower bounds when $p_1=0.5$ suggests that the value of Shannon entropy for Community A is not tightly constrained by its most abundant species. On the other hand, Community B, with a single dominant species, falls towards the right-hand side of Fig. 1G, with $p_1=0.85$. In general, large values of $p_1$ tightly constrain Shannon entropy; for each $n\geqslant 3$ in Fig. 1, the space between the upper and lower bounds at $p_1=0.5$ is larger than at $p_1=0.85$. Thus, because Community B has one dominant species, its Shannon entropy is almost exclusively determined by the relative abundance of that species, and the abundances of rarer species could change without substantially influencing the Shannon entropy. This effect—that the Shannon entropy is constrained by a dominant species—is most pronounced with small species richness *n*.

## Bounds on Shannon entropy: the *i*th-most abundant species

Now consider a community with multiple dominant species, rather than just one. How do the values of the second, third, or, in general, *i*th-most abundant species constrain Shannon entropy?

We now present our new bounds on Shannon entropy as a function of the *i*th-greatest species abundance. We saw in the $i=1$ case that entropy is maximized when abundances are as evenly distributed across the entries of the vector as possible. We construct the maximizing vector for $i\geqslant 2$ similarly, setting every species abundance before or after the *i*th equal to $p_i$; whether entries before or after the *i*th one equal $p_i$ depends on the length of the vector and the value of $p_i$, since the vector’s sum cannot be greater than 1. All remaining vector entries are set equal to each other.

In the $i=1$ case, we observed that entropy is minimized when as few species as possible possess non-zero abundances. We thus construct the minimizing vector by setting the abundances of the second through the *i*th species equal to $p_i$, placing all remaining weight in the first species.

The bounds are proven in Appendix A.

### Theorem 3

For a fixed value $p_i$ of the frequency of the *i*th-most abundant species in a community with *n* species, with $i \geqslant 2$, the vector maximizing *H* is $p^\prime$, where

$$\begin{aligned} p^\prime = {\left\{ \begin{array}{ll} \left( p_i, p_i, \ldots , p_i, \frac{1-ip_i}{n-i}, \frac{1-ip_i}{n-i}, \ldots , \frac{1-ip_i}{n-i}\right) , &{} p_i\geqslant \frac{1}{n}, \\ \left( \frac{1-(n-i+1)p_i}{i-1}, \frac{1-(n-i+1)p_i}{i-1}, \ldots , \frac{1-(n-i+1)p_i}{i-1}, p_i, p_i, \ldots , p_i\right) , &{} p_i < \frac{1}{n}. \end{array}\right. } \end{aligned}$$

In the $p_i\geqslant \frac{1}{n}$ case, *i* entries equal $p_i$. In the $p_i < \frac{1}{n}$ case, $i-1$ entries equal $\frac{1-(n-i+1)p_i}{i-1}$.

**Fig. 2:**
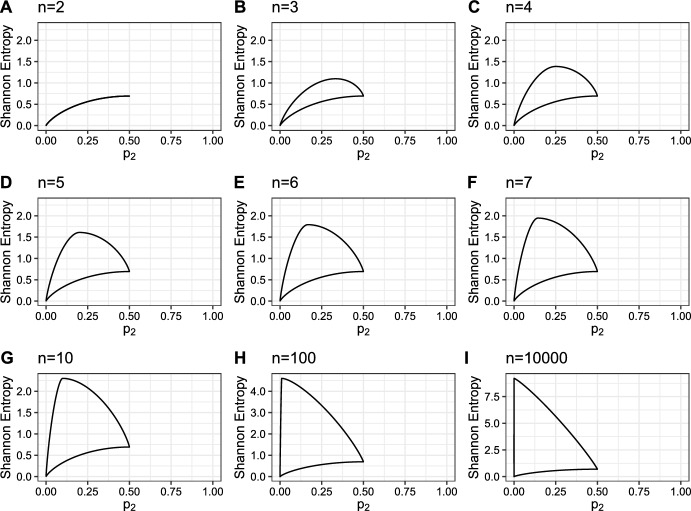
Upper and lower bounds on Shannon entropy from Theorems 3 and 4 as functions of the abundance of the second-most abundant species, $p_2$, for fixed *n*. **A** $n=2$. **B** $n=3$. **C** $n=4$. **D** $n=5$. **E** $n=6$. **F** $n=7$. **G** $n=10$. **H** $n=100$. **I** $n=10{,}000$. For fixed *n*, *H* is maximized when $p=(\frac{1}{n}, \frac{1}{n}, \ldots , \frac{1}{n})$, giving $H(\frac{1}{n},\frac{1}{n}, \ldots , \frac{1}{n})=\log n$. Thus, the point on every plot with the highest Shannon entropy lies at $(\frac{1}{n}, \log n)$. Because $p_2$ is the second-most abundant species, it cannot exceed $\frac{1}{2}$. Note that panels H and I have y-axis scales that differ from those of the other panels

The upper bound on *H* is

4 $$\begin{aligned} H(p^\prime )=H_{{\max }}(p_i, n)= {\left\{ \begin{array}{ll} i h(p_i)+ (n-i) \, h\Big (\frac{1-ip_i}{n-i}\Big ), &{} p_i \geqslant \frac{1}{n}, \\ (i-1) \, h\Big (\frac{1-(n-i+1)p_i}{i-1}\Big )+(n-i+1)\, h(p_i), &{} p_i < \frac{1}{n}. \end{array}\right. } \end{aligned}$$

### Theorem 4

For a fixed value $p_i$ of the frequency of the *i*th-most abundant species in a community with *n* species, with $i\geqslant 2$, the vector minimizing *H* is $p^{\prime \prime }$, where

$$\begin{aligned} p^{\prime \prime } = \big (1-(i-1)p_i, p_i, p_i, \ldots , p_i, 0, 0, \ldots , 0 \big ), \end{aligned}$$

and $i-1$ entries equal $p_i$. The lower bound on *H* is

5 $$\begin{aligned} H(p^{\prime \prime })=H_{\min }(p_i, n) = h\big (1-(i-1)p_i\big ) + (i-1)\, h(p_i). \end{aligned}$$

**Fig. 3:**
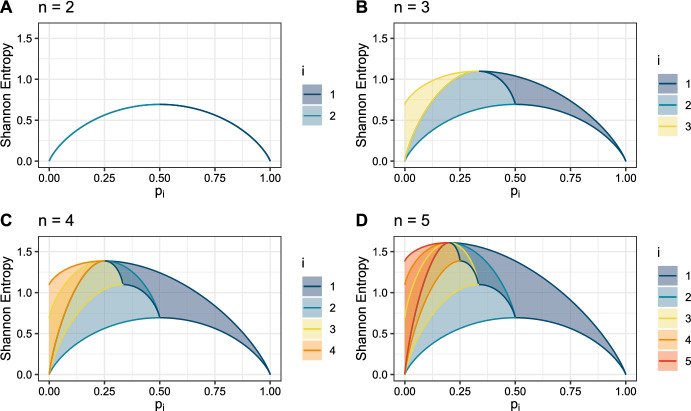
Upper and lower bounds on Shannon entropy as functions of the abundance of the *i*th-most abundant species, $p_i$, for fixed *n*. **A** $n=2$. **B** $n=3$. **C** $n=4$. **D** $n=5$. Lines give the upper and lower bounds from Theorems 3 and 4, colored according to which abundance, $p_i$, is fixed. The space between the upper and lower bounds for a given *i* is shaded based on the color used for the associated bounds

We explore these bounds visually in Figs. 2 and 3. Figure 2 gives the upper and lower bounds on Shannon entropy as a function of $p_2$, the abundance of the second-most abundant species, for varying vector lengths *n*. As in Fig. 1, the upper bound increases with increasing *n*, and the lower bound remains the same irrespective of the value of *n*. As $p_2$ increases toward its maximum of $\frac{1}{2}$, the Shannon entropy becomes tightly constrained, with the upper and lower bounds approaching the same point $(\frac{1}{2}, \log 2)$.

We examine the bounds on Shannon entropy conditional on the *i*th-largest entry for vectors of length $n=2$, 3, 4, and 5 in Fig. 3. For all four panels, the shapes outlined by the bounds for fixed $p_1$ are identical to the corresponding panels in Fig. 1, and the shapes outlined by the bounds for fixed $p_2$ match corresponding panels in Fig. 2. For all *i*, as *n* increases from $n=i$, the upper bound on *H* with respect to $p_i$ increases; the lower bound remains the same.

Examining the regions between the upper and lower bounds in the $(p_i,H)$-plane for distinct values of *i* with fixed *n*, we observe a number of patterns. For $n=2$, the regions overlap only trivially, at the point $(\frac{1}{2},\log 2)$ in Fig. 3A (Proposition B.1). For $n=3$, the overlap is also trivial, occurring along a curve; for $n=3$, the upper bound on Shannon entropy given $p_2$ overlaps exactly with the lower bound on Shannon entropy conditional on $p_3$ from 0 to $\frac{1}{3}$, then with the lower bound given $p_1$ from $\frac{1}{3}$ to $\frac{1}{2}$ (Fig. 3B, upper bound of the turquoise region; Proposition B.2). For fixed $n \geqslant 4$, regions for differing *i* begin to have nontrivial overlap. For $2 \leqslant i \leqslant n-2$, the region between the bounds conditional on $p_i$ overlaps with the region between the bounds conditional on $p_1$ (Fig. 3D, the turquoise and yellow regions overlap the navy region; Proposition B.6). The upper bound conditional on $p_{n-1}$ exactly overlaps with the lower bound conditional on $p_1$ for a small interval from $\frac{1}{n}$ to $\frac{1}{n-1}$ (Fig. 3D, the orange upper bound and the navy lower bound overlap between $\frac{1}{5}$ and $\frac{1}{4}$; Proposition B.7). On the left-hand side of each panel, we can see that when $p_i=0$, the intervals of possible Shannon entropy values for each pair of indices $i_1,i_2\geqslant 2$ overlap for $n \geqslant 3$; the overlap has nonzero length at $p_i=0$, except that for the pair of values $(i_1,i_2)=(2,n)$, the intervals overlap only at a single point (Proposition B.8). This overlap of the intervals for a pair of indices continues as $p_i$ increases; for the pair $(i_1,i_2)=(2,n)$, it is a curve rather than a region of nonzero area (Proposition B.9). We explain these observations mathematically in Appendix B.

## Data analysis

Having established upper and lower bounds on Shannon entropy as functions of the abundance of the *i*th-most abundant species, we turn to two data sets in order to explore applications of the bounds to communities with vastly differing numbers of taxa: one example has dozens of taxa, the other has tens of thousands. We then compare diversity between the two examples, exploring the extent to which knowledge of the bounds helps to inform comparisons of diversity between communities occupying different regions of the space of possible taxon abundances.

### Coral reefs

Wong et al. (2018) analyzed 25 coral reef communities sampled off the southern coast of Singapore. Among the communities, 18 are “fringe” reefs that border 11 offshore islands, 5 are offshore “patch” reefs that are exposed at low tide, and 2 are “regrowth” reefs growing on artificial structures. At each site, Wong et al. (2018) sampled five 20-meter transects, for each transect generating a vector of observed species abundances. The $25 \times 5=125$ vectors can each be normalized to produce relative abundance vectors that sum to 1. Across the 125 transects, 138 species were observed.

**Fig. 4:**
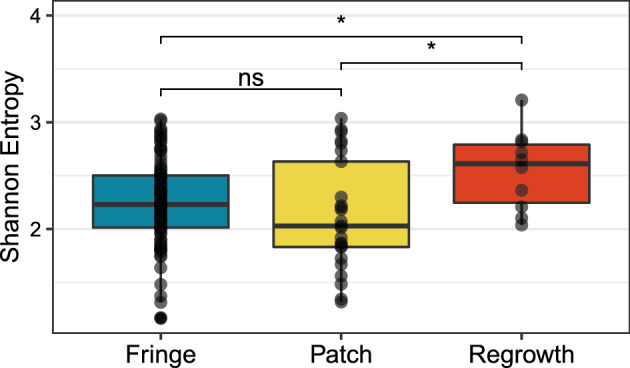
Distributions of Shannon entropy for three coral reef types. Each point represents a relative abundance vector of coral species along one transect. For each of 25 study sites, 5 transects were measured. The 25 sites include 18 “fringe” sites that border offshore islands, 5 offshore “patch” sites that are exposed at low tide, and 2 “regrowth” sites growing on artificial structures. Accordingly, there are $18\times 5=90$ fringe data points, $5\times 5=25$ patch data points, and $2\times 5=10$ regrowth data points. The three distributions differ in Shannon entropy (Kruskal–Wallis test, $P =0.037$). Regrowth reefs have significantly higher values of Shannon entropy than fringe or patch reefs (Wilcoxon rank sum test, $P<0.05$)

The species richness of transects varies from 6 to 31, with mean 15.3, median 14, and standard deviation 6.1. To compare the species diversity across transects, we computed the Shannon entropy of each transect’s species relative abundance vector. The Shannon entropy ranges from 1.1 to 3.2 across transects, with mean 2.2, median 2.2, and standard deviation 0.4. We observe in Fig. 4 that the regrowth reefs have significantly higher Shannon entropy than the patch reefs and fringe reefs (Wilcoxon rank sum test, two-tailed $P=0.028$ for regrowth vs. patch, $P =0.034$ for regrowth vs. fringe). No significant difference exists for patch and fringe reefs ($P =0.183$).

We saw previously that the Shannon entropy of a species composition vector is influenced by many features of the vector, including the species richness and the evenness of the community composition. For example, if a community has one abundant species, then its Shannon entropy is strongly constrained by the abundance of that species. What drives the elevated diversity of the regrowth reef community when compared to the other two reef types?

**Fig. 5:**
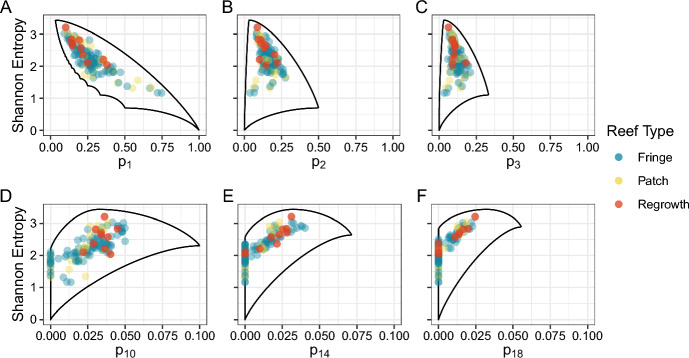
Upper and lower bounds on Shannon entropy for coral communities, as functions of the abundance of the *i*th-most abundant species. **A** $i=1$. **B** $i=2$. **C** $i=3$. **D** $i=10$. **E** $i=14$. **F** $i=18$. The bounds assume $n=31$, as 31 was the largest number of species observed across the 125 transects (mean 15.3, median 14, standard deviation 6.1, minimum 6). Bounds are computed according to Theorems 3 and 4. Each point represents one transect; points are colored according to reef type. As in Fig. 4, each panel shows 90 fringe data points, 25 patch data points, and 10 regrowth data points

Figure 5 depicts the relationship between each community’s Shannon entropy and the bounds on Shannon entropy conditional on various species abundances. Inspection of these relationships reveals a visual difference among the three reef types. Regrowth reefs appear to have lower abundances for more common species such as the 1st, 2nd, and 3rd most abundant (Fig. 5A–C) and higher abundances for rarer species such as the 10th, 14th, and 18th most abundant (Fig. 5D–F), allowing for higher values of Shannon entropy. A statistical test verifies this visual observation: comparing the abundance of the *i*th-most abundant species in regrowth reefs to that of non-regrowth reefs, we find that the frequencies of the 1st through 3rd most abundant species in regrowth reefs are lower than those in non-regrowth reefs (Wilcoxon rank sum test, $P = 0.029$ for $i=1$, $P=0.064$ for $i=2$, $P=0.036$ for $i=3$), whereas the 9th through 19th most abundant species are significantly greater in regrowth than non-regrowth reefs (Wilcoxon rank sum test, $P\leqslant 0.05$; Figure S1). This result suggests that the regrowth reefs have higher Shannon entropy values than the patch or fringe reefs in part because of their lower abundances of common species and higher abundances of rare species—which, in turn, owing to higher upper and lower bounds at those abundances, enable the Shannon entropy to reach higher values. Use of the bounds thus helps to illustrate that the rare species drive a difference in Shannon entropy across community types.

### Sponge microbiomes

We next analyzed microbial communities associated with 3533 sea sponges representing 24 distinct taxonomic orders, as sampled from 34 countries worldwide by Moitinho-Silva et al. (2017). For each sponge sample, Moitinho-Silva et al. (2017) amplified and sequenced the V4 region of the 16 s rRNA gene to generate a vector of abundances of microbial operational taxonomic units (OTUs). As with the coral data, we normalized each vector to generate relative abundance vectors.

**Fig. 6:**
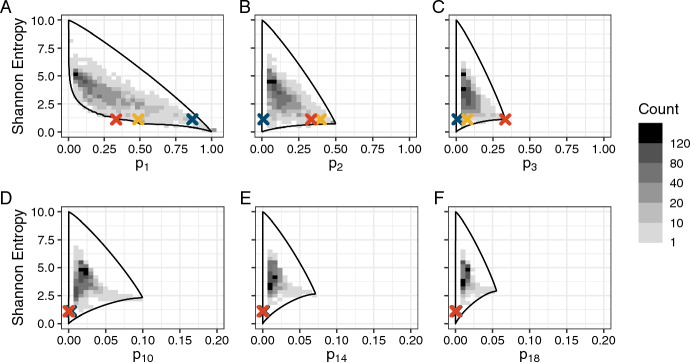
Upper and lower bounds on Shannon entropy for sponge microbiome communities, as functions of the abundance of the *i*th-most abundant OTU. **A** $i=1$. **B** $i=2$. **C** $i=3$. **D** $i=10$. **E** $i=14$. **F** $i=18$. The bounds assume $n = 21{,}595$, the largest number of OTUs observed across the 3533 microbiomes (mean 2230, median 1734, standard deviation 2072, minimum 1). Each count in the heat map represents one sampled sponge microbiome; the heat map summarizes 3533 microbiomes. The three highlighted points represent similar Shannon entropy $(H\approx 1.1\approx \log 3)$ but different evenness. Details on the three points appear in Table S1

Much variability exists in microbiome composition across the sampled sponges: OTU richness varies from 1 to 21,595, with mean 2230 and median 1734. We found a wide distribution of Shannon entropy values across microbiomes, ranging from 0 to 8.1, with mean 3.4, median 3.5, and standard deviation 1.3. In Fig. 6, we plot these values of Shannon entropy against the abundance of the *i*th-most abundant OTU for $i=1,2,3,10, 14, 18$.

To explore an application of the bounds on Shannon entropy in which multiple communities have similar Shannon entropy values rather than values that differ significantly, as in the corals, we highlight in Fig. 6 three microbial communities in red, yellow, and blue. These communities have similar Shannon entropy values: 1.10 for red, 1.11 for yellow, and 1.11 for blue (Table S1)—all near $H\big ( (\frac{1}{3}, \frac{1}{3}, \frac{1}{3})\big )=\log 3\approx 1.10$. Without knowledge of their relative abundance vectors, it is difficult to interpret the similarity in Shannon entropy: one community could have high evenness and low richness, another could have high richness but uneven abundances.

Examining *H* in relation to $p_i$ and the bounds on *H* in terms of $p_i$, we see that the three communities’ similar values of Shannon entropy are produced by quite different relative abundance vectors. Figure 6A shows that the red community has the lowest possible value of entropy given the abundance of its most abundant OTU. The yellow community is similarly positioned near the lower bound. The blue community, on the other hand, though it has a Shannon entropy that is intermediate between the lower and upper bounds, has a value of $p_1$ that tightly constrains the Shannon entropy. The tight constraint suggests that the entropy of the blue community is largely determined by the most abundant OTU, whereas the red and yellow communities depend on other entries of the relative abundance vector to keep the entropy near the lower bound.

Which other entries affect the entropy? In Fig. 6B, the horizontal ordering of the points has changed: the blue community, dominated by one abundant OTU, is near $p_2=0$, whereas the red and yellow communities have $p_2 \approx p_1$. Figure 6C identifies the abundance vector of the red community: the upper and lower bounds on Shannon entropy as a function of $p_3$ meet at $p_3 = \frac{1}{3}$, a point attained by exactly one relative abundance vector: $p = (\frac{1}{3}, \frac{1}{3}, \frac{1}{3})$. As the red community has $p_3=\frac{1}{3}$, this plot reveals that the red community has species richness $n=3$ and that its sampled community is evenly distributed. The blue community lies near $p_3=0$, and the yellow community also lies near $p_3=0$, suggesting that its community is largely dominated by just the first two OTUs.

An investigation of higher values of *i* (Fig. 6D–F) further illustrates that all three communities are dominated by their first few entries. The blue community continues to lie to the right of 0, with $p_{18}>0$ (Fig. 6F). In fact, the blue community has richness $n=1678$, but because $p_1$ is so high, in the part of the domain where Shannon entropy is tightly constrained, the subsequent entries after the first have little impact on entropy.

Considering the relationship between three vectors with similar Shannon entropy together with the Shannon entropy bounds as functions of $p_i$ has illustrated that the same Shannon entropy can indicate a low diversity conditional on a specified abundance ($p_1$ or $p_2$ for the red and yellow communities), or a highly constrained diversity largely determined by the first entry ($p_1$ for the blue community), despite the occurrence of many non-zero entries in the vector. Knowledge of the constraints thus aids with the interpretation of Shannon entropy across communities not only when entropy values are different, as for the coral example, but also when they are similar.

### Comparing the example data sets

**Fig. 7:**
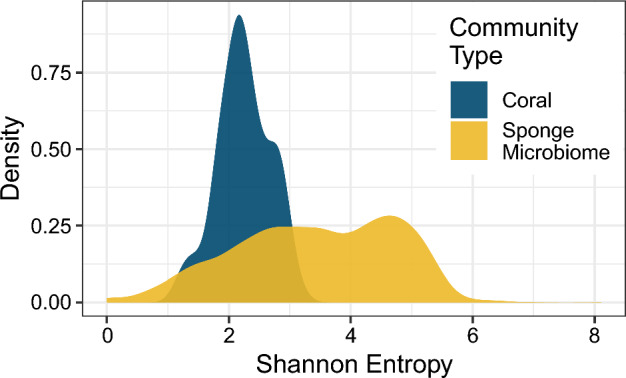
Distributions of Shannon entropy for coral (navy) and sponge microbiome communities (orange). There are 125 total coral communities and 3533 total sponge microbiomes represented. Entropy values for the coral communities are the same as those presented in Figs. 4 and 5, whereas those for the sponge microbiomes are the same as those presented in Fig. 6

We now illustrate the use of the entropy bounds in comparing communities across the two examples, as an example of how the bounds make quite different communities commensurable. Figure 7 plots distributions of Shannon entropy across communities for the two data sets. A simple interpretation of this comparison would conclude that, in terms of Shannon entropy, the coral communities are on average less diverse than the sponge microbiome communities, because they have significantly lower Shannon entropy (Wilcoxon rank sum test, $P<2.2\times 10^{-16}$). The richnesses in the two examples, however, differ by nearly three orders of magnitude ($n=31$ for corals, $n=21{,}595$ for sponge microbiomes). Because the upper bound on entropy as a function of *n* is $\log n$, the largest attainable value of entropy for a coral community is $\log 31 \approx 3.4$, whereas the corresponding maximum for a sponge microbiome is $\log 21{,}595 \approx 10.0$. How can we compare the entropies of communities that have such a large difference in taxon richness?

**Fig. 8:**
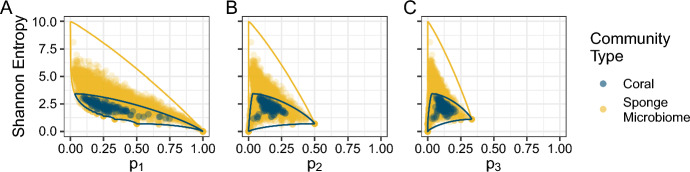
Bounds on Shannon entropy for coral (navy) and sponge microbiome communities (orange), as functions of the abundance of the *i*th-most abundant taxon. **A** $i=1$. **B** $i=2$. **C** $i=3$. The coral bounds assume $n=31$, and the sponge bounds assume $n=21{,}595$. Each point represents one sampled relative abundance vector. The points and bounds are the same as those presented in Fig. 5 for corals and Fig. 6 for sponge microbiomes

Consider plots of *H* in relation to $p_i$ for both data sets at once, along with the bounds on entropy with respect to the *i*th-most abundant taxon (Fig. 8). Although we see that the points for corals (navy) do have lower values of Shannon entropy than those for the sponge microbiomes (orange), the interpretation changes substantially. For the corals, the cloud of points is generally centered in the middle or top of the space between the bounds, with no points along the lower bound. Conversely, for the sponge microbiomes, the cloud of points consistently borders the lower bound, with few points near the upper bound. The sponge microbiome communities, though more diverse according to *H*, are not nearly as diverse as they could be given their high OTU richness; coral communities, on the other hand, are diverse given their relatively low species richness.

Note, however, that this conclusion is affected by our decision to use the maximal richness as the value of *n* for generating the bounds. Because the maximal Shannon entropy increases with *n*, many communities with richness less than the maximum cannot reach the upper bound because such a choice of *n* sets an upper bound that is higher than would be possible in those communities. If, on the other hand, we were to generate the bounds using a value of *n* that lies below the maximum, then we must either exclude samples with richnesses above *n* or truncate and renormalize them.

To probe this limitation, we performed two additional analyses. First, we reproduced Fig. 8 using the median coral species richness $n=14$ instead of the maximum $n=31$ and the median sponge microbiome OTU richness $n=1734$ instead of the maximum $n=21{,}595$ (Figure S2). We truncated vectors with more than 14 coral or 1734 sponge microbiome taxa to the 14 or 1734 most abundant taxa, renormalizing each vector so that its sum was still 1. Although upper bounds for both communities shift down slightly, the main observation from Fig. 8 remains visible: coral relative abundance vectors largely occupy a region squarely between the upper and lower bounds—or even closer to the upper bound than the lower—whereas sponge microbiome relative abundance vectors reach the lower but not the upper bound. Thus, even with a change in the way species richness is considered in obtaining the bounds, the coral communities, despite lower numerical entropy values, are nearer to their upper bounds on entropy than are the sponge microbiomes.

**Fig. 9:**
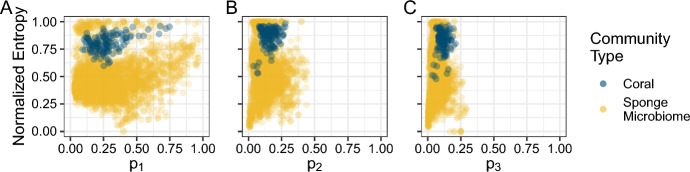
Shannon entropy for coral (navy) and sponge microbiome communities (orange), normalized in relation to entropy bounds as functions of the abundance of the *i*th-most abundant taxon. **A** $i=1$. **B** $i=2$. **C** $i=3$. Each point represents one sampled relative abundance vector, with Shannon entropy normalized using Eq. 6

**Fig. 10:**
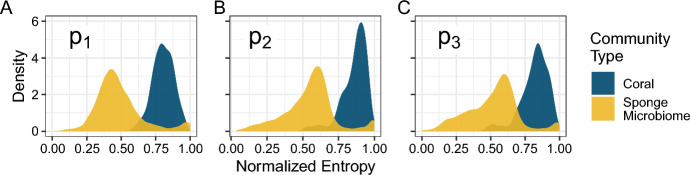
Distributions of normalized Shannon entropy for coral (navy) and sponge microbiome communities (navy) normalized in relation to entropy bounds as a function of the *i*th-most abundant species. **A** $i=1$. **B** $i=2$. **C** $i=3$. The plots summarize the distributions of normalized Shannon entropy presented in Fig. 9

For our second approach to addressing the role of species richness in entropy bounds, we normalized each vector’s entropy using bounds given that vector’s length, *n*, and the chosen *i*:

6 $$\begin{aligned} H_\text {norm}(p, n; i) = \frac{H(p) - H_{\min }(p_i, n)}{H_{\max }(p_i, n) - H_{\min }(p_i, n)}. \end{aligned}$$

This approach ensures that the bound used for a community is suited to the richness of that community. It is unsuitable only in rare cases such as the red community in Fig. 6 in which $p_i = \frac{1}{i}$ and the abundance vector of a community is determined by a single abundance—so that upper and lower bounds are equal and Eq. 6 has denominator zero. We exclude such communities from our calculation.

Figure 9 presents the normalized Shannon entropy of Eq. 6 versus $p_1$, $p_2$, and $p_3$ for the coral and sponge microbiome data. When each vector is normalized using bounds that account for that vector’s length, *n*—and not a possibly larger value chosen to represent a collection of vectors, as in Fig. 8—the points fall closer to the upper bound. In particular, many of the sponge microbiome relative abundance vectors have normalized entropy values equal to 1, whereas none reached the upper bound in Fig. 8. Despite this trend, the coral communities consistently have significantly higher normalized Shannon entropy than the sponge microbiome communities (Fig. 10; Wilcoxon rank sum test, $P<2.2\times 10^{-16}$ for normalizations based on $p_1$, $p_2$, or $p_3$), a reversal of the pattern for unnormalized entropy (Fig. 7). This statistical result formalizes our observation that, despite having lower absolute values of Shannon entropy than the sponge microbiome communities, the coral communities’ values of Shannon entropy lie closer to their upper bounds.

## Discussion

We have explored the range of Shannon entropy values that can be attained by a frequency vector of specified length and fixed *i*th-largest entry—such as a vector of species relative abundances in a community. Our upper and lower bounds on Shannon entropy as a function of the number of species, *n*, and the abundance of the *i*th most common species, $p_i$, characterize the relationship between Shannon entropy and $p_i$, providing insight into the way in which entropy values are constrained by the abundances of more abundant or less abundant species.

Our main mathematical results, Theorems 3 and 4, generalize a previous result of Aw and Rosenberg (2018) on the Shannon entropy bounds with respect to $p_1$, the abundance of the most abundant species. For each $p_i$, $2 \leqslant i \leqslant n$, the permissible region given subsequent abundance $p_i$ contains points $(\frac{1}{n},\log n)$ and $(\frac{1}{i},\log i)$ (Fig. 3). Unlike for $p_1$, the permissible region for $p_i$, $i\geqslant 2$, contains the origin; the permissible region for $p_1$ instead contains the point (1, 0) (Fig. 1). The extension to $p_i$ for $i \geqslant 2$ characterizes a new set of similar regions—examined in detail in Appendix B—that differ substantially from the region previously studied for $i=1$.

To illustrate the utility of the mathematical results for studies of biodiversity, we considered them in two data sets. In coral communities, we found that the higher Shannon entropy of regrowth reefs, as opposed to patch or fringe reefs (Fig. 4), was driven by low abundances of common taxa and high abundances of rare taxa (Fig. 5). If $p_1$ is large, then the upper bound on Shannon entropy given $p_1$ is low, so entropy must be low (Fig. 1); conversely, if subsequent $p_i$ are large, then the lower bound on Shannon entropy given $p_i$ is high, so entropy must be relatively large (Fig. 3D). Because common species had relatively low abundances in the regrowth reefs, the communities occupied a region of $(p_i, H)$-space that for small *i* was not tightly constrained by $p_i$, allowing them to achieve high values of entropy (Fig. 5A-C). Similarly, for larger *i*, the relatively large abundances of rare species in the regrowth reefs placed them in a region of $(p_i, H)$-space with high lower bounds, requiring these communities to have fairly high entropy (Fig. 5D-F). By visualizing the abundance of a fixed species in a community in relation to that community’s Shannon entropy and its bounds, we were able both to identify differences between types of communities and to uncover properties of the communities that drove the differences.

In our analysis of corals, we considered communities with differing values of Shannon entropy; our second analysis, examining sponge microbiomes, considered communities with similar Shannon entropy values, despite quite different taxon abundance distributions. By studying three example communities’ Shannon entropy values relative to the bounds on entropy, we identified key differences among the abundance vectors (Fig. 6). Whereas one community’s entropy was strongly constrained by its large $p_1$ and lay close to its upper bound, the entropy values of the other two were near their minima given $p_1$ (Fig. 6A). The similarity of the entropy values in these other communities was achieved by a low diversity among the subsequent taxa for communities with relatively low $p_1$, as can be seen from the fact that these communities have entropy values that are also quite constrained in relation to $p_2$, $p_3$, or both (Fig. 6B,C). The bounds thus assist in explaining not only differences in entropy values across communities, but also similarities.

Finally, in our comparative analysis of the two example data sets, we demonstrated that knowledge of the bounds on entropy was useful for accurately interpreting differences in Shannon entropy distributions between the coral and sponge microbiome communities, which differ greatly in species richness (Fig. 8). A naive comparison of the Shannon entropies of the two community types suggested that the sponge microbiomes were more diverse than the corals (Fig. 7). However, when the bounds were considered, either visually (Fig. 8) or via normalization of the Shannon entropy by use of the bounds (Figs. 9 and 10), the coral communities were more diverse given the constraint of their species richness and the abundances of one of their more abundant species.

All these analyses address a challenge in comparing the diversity of communities with different numbers of species. Similarities in a single statistic such as Shannon entropy can obscure meaningful compositional differences or species richness differences between communities. Our mathematical results assist in understanding how Shannon entropy is constrained by individual abundances, enabling comparisons both through visually analyzing Shannon entropy in relation to the bounds and through computations of the normalized entropy in Eq. 6 (Fig. 10). In addition to the bounds of Aw and Rosenberg (2018) on Shannon entropy in terms of $p_1$, the general upper bound of $\log n$ over all abundance vectors has long been used in normalizations (Pielou 1975, p. 15); a special case of a lower bound for all abundance vectors in finite samples with fixed species richness and sample size has also appeared in a normalization as well (Beisel and Moreteau 1997). Our normalization uses the tightest possible interval for any fixed $p_i$, showing that for any $p_i \ne \frac{1}{n}$, Shannon entropy has a tighter upper bound than $\log n$—and it has a non-zero lower bound as well. The transformation in Eq. 6 follows a form familiar from other normalizations (e.g. Beisel and Moreteau 1997; Jost 2010).

Because Shannon entropy is used often, mathematical properties of this statistic have implications for many routine ecological analyses. In particular, researchers reporting Shannon entropy might be advised to report not only the Shannon entropy, but also its upper and lower bounds in relation to the highest or subsequent abundances, so that the Shannon entropy value can be further contextualized. Such an approach has been suggested for a variety of diversity-related statistics in population genetics, for which mathematical bounds in relation to allele frequencies have been analogously reported (e.g. Maruki et al. 2012; Jakobsson et al. 2013; Garud and Rosenberg 2015; Alcala and Rosenberg 2017, 2019, 2022).

Our examples have demonstrated the value of the bounds in understanding the drivers of empirical differences in biodiversity between communities. However, the bounds might also be useful in efforts to test model-based theoretical predictions about species abundances (e.g. Chave 2004; Rosindell et al. 2011). For example, in tests of neutral and other models describing rare taxa in a community (e.g. Magurran and Henderson 2003), versions of Shannon entropy normalized by the bounds conditional on the abundances of common taxa could control for those abundances, serving as biodiversity metrics sensitive to the abundances of rare taxa.

The data analyses in corals and sponge microbiomes follow population-genetic studies such as Aw and Rosenberg (2018) in treating quantities measured in samples as parametric. A potential extension could incorporate the fact that both the relative abundances and the number of distinct species itself are measured in samples. Equation 12 of Alcala and Rosenberg (2017) described related bounds on an estimated value of the population-genetic statistic $F_{ST}$ in terms of a sample frequency. In that setting, the number of alleles was fixed at 2, but here, the number of distinct species in a sample underestimates the number in the full community, so that the permissible range for the estimated Shannon entropy might systematically expand—due to the increased number of species—as the sample is enlarged. An extension to the bounds that incorporates sampling phenomena might make use of approaches to estimation of Shannon entropy in the setting of species accumulation with increasing sample size (Chao and Shen 2003; Chao and Jost 2015).

Although our use of Shannon entropy has focused on biodiversity measurement, the mathematical results are broader in scope. First, although we have used the language of species abundances, the entropy bounds apply to any finite-length vectors of non-negative elements that sum to 1, and are thus not limited to ecological abundance data. The bounds could have uses for other settings in which Shannon entropy is used as a diversity statistic, such as for other taxonomic levels or for population-genetic data (Sherwin et al. 2006, 2017; Aw and Rosenberg 2018). They contribute to an interdisciplinary body of work developing bounds on Shannon entropy in various contexts (e.g. Dembo et al. 1991; Berry and Sanders 2003; Khan et al. 2017).

Second, we have obtained our mathematical bounds on Shannon entropy as a corollary of general theorems that concern statistics with particular convexity properties (Appendix A). Related statistics such as the Rényi entropies or Hill numbers (Hill 1973; Jost 2006; Chao et al. 2014) possess the required properties, so that similar bounds will follow for these statistics in relation to the abundance of the *i*th most abundant species. Extensions could explore constraints on these statistics and the applications of the constraints to ecological data.

### Supplementary Information

Below is the link to the electronic supplementary material.

Supplementary file 1 (pdf 800 KB)

## Data Availability

The study uses previously published publicly available data. Coral reef data from Wong et al. (2018) were downloaded from https://zenodo.org/record/1197411. Sponge microbiome data from Moitinho-Silva et al. (2017) were downloaded from http://gigadb.org/dataset/view/id/100332. An R script implementing our bounds on Shannon entropy is available at github.com/MaikeMorrison/EntropyBounds.

## Appendix A

This appendix proves the upper and lower bounds on Shannon entropy as functions of the *i*th-largest entry of a vector (Theorems 3 and 4). The proof relies on *majorization* (Marshall et al. 2010), a framework that orders vectors by a sense of their levels of concentration and that has sometimes been used in an ecological context (Patil and Taillie 1982; Mosler 2001; Liu et al. 2007).

### Definition A.1

Consider two vectors *w* and *v* of length *n* whose elements are in decreasing order, $w_i \geqslant w_j$ and $v_i \geqslant v_j$ for $i < j$. Vector *w* is said to **majorize** *v* if

(i) $\sum _{i=1}^k w_i \geqslant \sum _{i=1}^k v_i$ for all $k<n$, and
(ii) $$\sum _{i=1}^n w_i = \sum _{i=1}^n v_i$$.

If *w* majorizes *v*, then we write $w\succ v$ or $v \prec w$.

Note that we assume here that entries of *w* and *v* are in decreasing order. For two vectors that are not necessarily in decreasing order, *w* is also said to majorize *v* if, when the vector entries are permuted so that they are in decreasing order, condition (i) holds for the permuted vectors.

We begin by proving that vector $p^\prime$ introduced in the statement of Theorem 3 is majorized by all other vectors with *i*th-largest entry $p_i$ (Lemma A.2). Next, we prove that the vector $p^{\prime \prime }$ majorizes all other vectors with *i*th-largest entry $p_i$ (Lemma A.3). We then need only introduce the idea of functions that preserve order under majorization before we can quickly obtain Theorems 3 and 4.

### Lemma A.2

Consider a non-negative vector *w* of length *n* with (i) entries in decreasing order, $w_i \geqslant w_j$ for $i < j$, (ii) $\sum _{i=1}^n w_i = 1$, and (iii) for some $i\geqslant 2$, the *i*th largest entry in *w*, $w_i$, is equal to $p_i$. Then *w* majorizes $p^\prime$.

### Proof

Let *w* be a vector of length *n* whose entries are in decreasing order, the sum of whose entries is 1, and whose *i*th component equals $p_i$. Then $w_j \geqslant p_i$ for all $j\leqslant i$, $w_j\leqslant p_i$ for all $j\geqslant i$, and $w_i = p_i$. Condition (ii) of Definition A.1 is satisfied, as *w* and $p^\prime$ both have sum 1. To verify condition (i), we break the problem into two cases.

(1) Suppose $p_i\geqslant \frac{1}{n}$. Because $w_j \geqslant p_i$ for all $j\leqslant i$, for all *k* in [1, *i*], $\sum _{j=1}^k w_j \geqslant k p_i= \sum _{j=1}^k p^{\prime }_j$. It remains to show that for all *k* in $[i+1,n]$, $\sum _{j=1}^k w_j \geqslant \sum _{j=1}^k p^{\prime }_j$.

Suppose for contradiction that some value of *k* in $[i+1,n-1]$ has $\sum _{j=1}^k w_j < \sum _{j=1}^k p^{\prime }_j$. This *k* has

$$\begin{aligned} \sum _{j=1}^k w_j = \bigg ( \sum _{j=1}^{i} w_j \bigg ) + \bigg ( \sum _{j=i+1}^k w_j \bigg ) < \sum _{j=1}^k p^{\prime }_j = ip_i + (k-i)\bigg (\frac{1-ip_i}{n-i}\bigg ). \end{aligned}$$

We have already shown that $\sum _{j=1}^i w_j \geqslant ip_i$. We are left with

$$\begin{aligned} ip_i + \sum _{j=i+1}^k w_j \leqslant \bigg ( \sum _{j=1}^i w_j \bigg ) + \bigg ( \sum _{j=i+1}^k w_j \bigg ) < ip_i + (k-i)\bigg (\frac{1-ip_i}{n-i}\bigg ), \end{aligned}$$

which implies that

$$\begin{aligned} \sum _{j=i+1}^k w_j < (k-i)\bigg (\frac{1-ip_i}{n-i}\bigg ). \end{aligned}$$

Dividing by $k-i$, the mean of the $w_j$ for *j* in $[i+1,k]$ is less than $(1-ip_i)/(n-i)$. Because the $w_j$ are in decreasing order, $w_k$, the smallest of the $w_j$ for *j* in $[i+1,k]$, satisfies $w_k < (1-ip_i)/(n-i)$.

As a result,

$$\begin{aligned} \sum _{j=k+1}^n w_j < (n-k) \bigg ( \frac{1-ip_i}{n-i} \bigg ). \end{aligned}$$

It follows that

$$\begin{aligned} \sum _{j=1}^k w_j = 1- \sum _{j=k+1}^n w_j > 1 - (n-k) \bigg ( \frac{1-ip_i}{n-i} \bigg ) = \sum _{j=1}^k p_j^\prime , \end{aligned}$$

a contradiction of $\sum _{j=1}^k w_j < \sum _{j=1}^k p_j^\prime$. We conclude that for all *k* in $[i+1,n]$, $\sum _{j=1}^k w_j \geqslant \sum _{j=1}^k p^{\prime }_j$. As we already showed that $\sum _{j=1}^k w_j \geqslant \sum _{j=1}^k p^{\prime }_j$ for all *k* in [1, *i*], we have proven $w \succ p^{\prime }$.

(2) Suppose $p_i < \frac{1}{n}$. Consider a value *k* in $[1,i-1]$. Suppose for contradiction that $w_k < \frac{1-(n-i+1)p_i}{i-1}$. Because $w_i \geqslant w_j$ for $i < j$, it follows that

$$\begin{aligned} \sum _{j=1}^k w_j < k \bigg [\frac{1 - (n-i+1)p_i}{i-1}\bigg ] = \sum _{j=1}^k p^{\prime }_j. \end{aligned}$$

We also have $w_j \leqslant w_k < \frac{1-(n-i+1)p_i}{i-1}$ for *j* in $[k+1,i-1]$, and $w_j \leqslant p_i$ for *j* in [*i*, *n*]. Then

$$\begin{aligned} \sum _{j=1}^n w_j < (i-1) \bigg [\frac{1 - (n-i+1)p_i}{i-1}\bigg ] + (n-i+1)p_i = 1, \end{aligned}$$

a contradiction of $\sum _{j=1}^n w_j = 1$. We have shown that for *k* in $[1,i-1]$, $w_k \geqslant \frac{1-(n-i+1)p_i}{i-1}$. Hence,

A1 $$\begin{aligned} \sum _{j=1}^k w_j \geqslant \sum _{j=1}^k p^{\prime }_j. \end{aligned}$$

Setting $k=i-1$, we add $w_i = p^{\prime }_i = p_i$ to both sides of Eq. A1, obtaining $\sum _{j=1}^i w_j \geqslant \sum _{j=1}^i p^{\prime }_j$.

For *j* in $[i+1,n]$, $p^{\prime }_j = p_i$. Hence, for *k* in $[i+1,n]$,

$$\begin{aligned} \sum _{j=1}^k p^{\prime }_j = (i-1)\bigg [\frac{1 - (n-i+1)p_i}{i-1}\bigg ] + (k-i+1)p_i = 1 - (n-k)p_i. \end{aligned}$$

Suppose for contradiction that $\sum _{j=1}^k w_j < \sum _{j=1}^k p^{\prime }_j$ for some *k* in $[i+1,n]$. Then $\sum _{j=1}^k w_j < 1 - (n-k)p_i$. We then have $\sum _{j=k+1}^n w_j = 1 - \sum _{j=1}^k w_j > (n-k)p_i$: a sum of $n-k$ terms exceeds $(n-k)p_i$, so that the largest of the terms exceeds $p_i$. We have reached a contradiction of the decreasing order of the entries in *w*, as $w_{k+1}, w_{k+2}, \ldots , w_n$ are all bounded above by $w_i = p_i$. We conclude $\sum _{j=1}^k w_j \geqslant \sum _{j=1}^k p^{\prime }_j$ for all *k* in $[i+1,n]$.

As we already showed that $\sum _{j=1}^k w_j \geqslant \sum _{j=1}^k p^{\prime }_j$ for all *k* in [1, *i*], we have shown that for all *k* in [1, *n*], $\sum _{j=1}^k w_j \geqslant \sum _{j=1}^k p^{\prime }_j$, and therefore $w \succ p^{\prime }$. $\square$

### Lemma A.3

Consider a non-negative vector *v* of length *n* with (i) entries in decreasing order, $v_i \geqslant v_j$ for $i < j$, (ii) $\sum _{i=1}^n v_i = 1$, and (iii) for some $i \geqslant 2$, the *i*th-largest entry in *v*, $v_i$, is equal to $p_i$. Then *v* is majorized by $p^{\prime \prime }$.

### Proof

We first show that $p^{\prime \prime }_1 = 1 - (i-1)p_i \geqslant v_1$. Because $v_i=p_i$, $v_j \geqslant p_i$ for all $j\leqslant i$ and $v_j\leqslant p_i$ for all $j\geqslant i$. Consequently, $\sum _{j=2}^i v_j \geqslant (i-1)p_i$, so that $v_1 \leqslant 1 - \sum _{j=2}^i v_j \leqslant 1 - (i-1)p_i$.

Next, suppose for contradiction that for some *k* in $[2,i-1]$, $\sum _{j=1}^k v_j > \sum _{j=1}^k p^{\prime \prime }_j = 1-(i-1)p_i + (k-1)p_i$. We then have $\sum _{j=k+1}^i v_j < 1 - [1-(i-k)p_i] = (i-k)p_i$, from which the mean of $v_{k+1},v_{k+2}, \ldots , v_i$ is less than $p_i$. Because $v_i$ is the smallest of $v_{k+1},v_{k+2}, \ldots , v_i$, we have $v_i < p_i$, a contradiction of $v_i=p_i$. Therefore, we have shown that $\sum _{j=1}^k v_j \leqslant \sum _{j=1}^k p^{\prime \prime }_j$ for all *k* in $[1,i-1]$.

For *k* in [*i*, *n*], $\sum _{j=1}^k p^{\prime \prime }_j = 1$, so it is trivially true that $\sum _{j=1}^k v_j \leqslant \sum _{j=1}^k p^{\prime \prime }_j$. We have thus shown that $\sum _{j=1}^k v_j \leqslant \sum _{j=1}^k p^{\prime \prime }_j$ for all *k* in [1, *n*]. We conclude that $v \prec p^{\prime \prime }$. $\square$

We now apply the lemmas. For convenience, we denote by $\Delta _{n-1}$ the set of non-negative vectors of length *n* with sum equal to 1.

### Definition A.4

Consider a function $F:\Delta _{n-1} \mapsto \mathbb {R}$ so that for all vectors *w* and *v* with $w\succ v$, $F(w) \geqslant F(v)$. Such a function is said to be **Schur-convex**. If instead, for all vectors *w* and *v* with $w\succ v$, $F(w) \leqslant F(v)$, *F* is said to be **Schur-concave**.

A Schur-convex function preserves the ordering of the vectors in $\Delta _{n-1}$ under majorization. A Schur-concave function reverses the ordering.

### Theorem A.5

Consider a function $F:\Delta _{n-1} \mapsto \mathbb {R}$. If *F* is Schur-concave, then the vector $p^{\prime }$ maximizes *F* over the subset of $\Delta _{n-1}$ with *i*th-largest entry equal to $p_i$. If *F* is Schur-convex, then $p^{\prime }$ minimizes *F* over the subset of $\Delta _{n-1}$ with *i*th-largest entry equal to $p_i$.

### Proof

By Lemma A.2, the vector $p^{\prime }$ is majorized by every vector $w\in \Delta _{n-1}$ with fixed $p_i$. That is, for all *w* in $\Delta _{n-1}$ with *i*th-largest entry equal to $p_i$, $w\succ p^{\prime }$. By definition of Schur-concavity and Schur-convexity, if *F* is Schur-concave, then $F(w)\leqslant F(p^{\prime })$ for all such *w*, and $p^{\prime }$ maximizes *F*. If *F* is Schur-convex, then $F(w)\geqslant F(p^{\prime })$ for all such *w*, and $p^{\prime }$ minimizes *F*. $\square$

### Theorem A.6

Consider a function $F:\Delta _{n-1} \mapsto \mathbb {R}$. If *F* is Schur-concave, then the vector $p^{\prime \prime }$ minimizes *F* over the subset of $\Delta _{n-1}$ with *i*th-largest entry equal to $p_i$. If *F* is Schur-convex, then $p^{\prime \prime }$ maximizes *F* over the subset of $\Delta _{n-1}$ with *i*th-largest entry equal to $p_i$.

### Proof

By Lemma A.3, the vector $p^{\prime \prime }$ majorizes every vector $v\in \Delta _{n-1}$ with fixed $p_i$. That is, for all *v* in $\Delta _{n-1}$ with *i*th-largest entry equal to $p_i$, $v\prec p^{\prime \prime }$. By definition of Schur-concavity and Schur-convexity, if *F* is Schur-concave, then $F(v)\geqslant F(p^{\prime \prime })$ for all such *v*, and $p^{\prime \prime }$ minimizes *F*. If *F* is Schur-convex, then $F(v)\leqslant F(p^{\prime \prime })$ for all such *v*, and $p^{\prime \prime }$ maximizes *F*. $\square$

### Proof of Theorem 3

Shannon entropy is Schur-concave (Marshall et al. 2010, pp. 101, 562). By Theorem A.5, vector $p^{\prime }$ is the vector in $\Delta _{n-1}$ with fixed $p_i$ that maximizes Shannon entropy. $\square$

### Proof of Theorem 4

Shannon entropy is Schur-concave (Marshall et al. 2010, pp. 101, 562). By Theorem A.6, vector $p^{\prime \prime }$ is the vector in $\Delta _{n-1}$ with fixed $p_i$ that minimizes Shannon entropy. $\square$

## Appendix B

In this appendix, we provide mathematical proofs of informal claims from the main text about the bounds, as observed in Fig. 3. As in Appendix A, $\Delta _{n-1}$ denotes the set of non-negative vectors of length *n* with sum equal to 1.

### Proposition B.1

Consider vectors *p* in $\Delta _1$ with $p_1 \geqslant p_2$. (i) For $i=1$ and $i=2$, $H_{\min }(p_i,2)=H_{\max }(p_i,2)$. (ii) The bound functions $H_{\min }(p_1,2)=H_{\max }(p_1,2)$ and $H_{\min }(p_2,2)=H_{\max }(p_2,2)$ are equal if and only if $(p_1,H)=(p_2,H)=(\frac{1}{2}, \log 2)$.

### Proof

(i) Because $p_1 = 1- p_2$, fixing one of the $p_i$ determines the other and the Shannon entropy: $H(p_i) = -p_i\log p_i - (1-p_i)\log (1-p_i)$. Thus, Shannon entropy has only one possible value for vectors of length 2 with one fixed entry, and the upper and lower bounds given $p_i$ exactly overlap.

(ii) Because $p_1 \geqslant p_2$, the domain for $p_1$ is $[\frac{1}{2},1]$ and the domain for $p_2$ is $[0,\frac{1}{2}]$. At $p_1=p_2=\frac{1}{2}$, $H_{\min }(p_1,2)=H_{\max }(p_1,2)=H_{\min }(p_2,2)=H_{\max }(p_2,2)=(\frac{1}{2},\log 2)$.$\square$

### Proposition B.2

Consider vectors *p* in $\Delta _2$ with $p_1 \geqslant p_2 \geqslant p_3$. (i) $H_{\max }(p_2,3)$ for $p_2$ in $[0,\frac{1}{3}]$ is equal to $H_{\min }(p_3,3)$ for $p_3$ in $[0,\frac{1}{3}]$. (ii) $H_{\max }(p_2,3)$ for $p_2$ in $[\frac{1}{3}, \frac{1}{2}]$ is equal to $H_{\min }(p_1,3)$ for $p_1$ in $[\frac{1}{3}, \frac{1}{2}]$.

### Proof

(i) For fixed $p_3$ in $[0,\frac{1}{3}]$, Shannon entropy is minimized at $(1-2p_3, p_3, p_3)$ (Theorem 4). In this same interval for $p_2$, Shannon entropy is maximized at the vector $(1-2p_2, p_2, p_2)$ (Theorem 3). These vectors are the same when the same value is inserted for the lone free variable, confirming the exact overlap of the upper bound given $p_2$ and the lower bound given $p_3$ on the interval $[0,\frac{1}{3}]$.

(ii) In $[\frac{1}{3},\frac{1}{2}]$, the vector minimizing Shannon entropy given fixed $p_1$ is $(p_1, p_1, 1-2p_1)$ (Proposition 2), and the vector maximizing Shannon entropy given fixed $p_2$ is $(p_2, p_2, 1-2p_2)$ (Theorem 3). These vectors are identical when the same value is inserted for the lone free variable, confirming the exact overlap of the upper bound given $p_2$ and the lower bound given $p_1$ on the interval $[\frac{1}{3},\frac{1}{2}]$. $\square$

### Proposition B.3

Let $n \geqslant 2$. Consider vectors *p* in $\Delta _{n-1}$ with $p_1 \geqslant p_2 \geqslant \ldots \geqslant p_n$. On the interior of the domain $[\frac{1}{n},1]$, $H_{\min }(p_1,n)$ has $n-2$ points where it is not differentiable, falling at values $(p_1,H) = (\frac{1}{j}, \log j)$ for $2 \leqslant j \leqslant n-1$.

### Proof

From Proposition 2, $H_{\min }(p_1,n)$ contains a ceiling function $\lceil 1/p_1 \rceil$, which is discontinuous on the interior of the domain $[\frac{1}{n},1]$ at the $n-2$ points $\frac{1}{n-1}, \frac{1}{n-2}, \ldots , \frac{1}{2}$. In particular, approaching points $\frac{1}{j}$ for $j=2,3, \ldots , n-1$ from below, $\lceil 1/p_1 \rceil = j+1$, and approaching from above, $\lceil 1/p_1 \rceil = j$.

$H_{\min }(p_1,n)$ is differentiable on open intervals $(\frac{1}{n}, \frac{1}{n-1}), (\frac{1}{n-1}, \frac{1}{n-2}), \ldots , (\frac{1}{2},1)$. At the interval boundaries, $H_{\min }(p_1,n)$ is continuous: both $\lim _{p_1 \rightarrow {1}/{j}^-} H_{\min }(p_1,n)$ and $\lim _{p_1 \rightarrow {1}/{j}^+} H_{\min }(p_1,n)$ equal $\log j$. However, $\lim _{p_1 \rightarrow {1}/{j}^-} dH_{\min }(p_1,n)/dp_1 = -\infty$ and $\lim _{p_1 \rightarrow {1}/{j}^+} dH_{\min }(p_1,n)/dp_1 = 0$, so that $H_{\min }(p_1,n)$ is not differentiable at points $\frac{1}{j}$ for $j=2,3, \ldots n-1$. $\square$

### Proposition B.4

Let $i \geqslant 2$. For $n \geqslant i$, consider vectors *p* in $\Delta _{n-1}$ with $p_1 \geqslant p_2 \geqslant \ldots \geqslant p_n$. $H_{\max }(p_i,n)=H_{\min }(p_i,n)$ at the point $(p_i,H)=(\frac{1}{i}, \log i)$.

### Proof

Computing $H_{\max }(p_i,n)$ and $H_{\min }(p_i,n)$ at $p_i = \frac{1}{i}$, $\log i$ is obtained for both quantities. $\square$

### Proposition B.5

Let $i \geqslant 2$. For $n \geqslant i$, consider vectors *p* in $\Delta _{n-1}$ with $p_1 \geqslant p_2 \geqslant \ldots \geqslant p_n$. Both $H_{\max }(p_i,n)$ and $H_{\min }(p_1,n)$ pass through the point $(\frac{1}{n},\log n)$.

### Proof

This lemma is verified by computing $H_{\max }(p_i,n)$ at $p_i = \frac{1}{n}$ and $H_{\min }(p_1,n)$ at $p_1 = \frac{1}{n}$. $\square$

### Proposition B.6

Let $n \geqslant 4$. Consider vectors *p* in $\Delta _{n-1}$ with $p_1 \geqslant p_2 \geqslant \ldots \geqslant p_n$. Consider *i* with $2 \leqslant i \leqslant n-2$. On the interval $(\frac{1}{n},\frac{1}{i})$, $H_{\max }(p_i,n) > H_{\min }(p_1,n)$.

### Proof

The vector that maximizes Shannon entropy for fixed $p_i = x$ with $\frac{1}{n}< x < \frac{1}{i}$ and $i\geqslant 2$ is $p'(x,i,n)=(x, x, \ldots , x, \frac{1 - ix}{n-i}, \frac{1 - ix}{n-i}, \ldots , \frac{1 - ix}{n-i})$, where *i* entries equal *x* (Theorem 3). By definition, the point $\Big (x, H\big (p'(x,i,n)\big )\Big )$ lies on $H_{\max }(p_i=x,n)$, the upper bound conditional on fixed $p_i=x$, for values of *x* in the interval $\frac{1}{n}< x< \frac{1}{i}$.

This vector $$p'(x,i,n)$$ also lies in the space of permissible vectors with fixed frequency $$p_1 = x$$. Further, $$p'(x,i,n)$$ is not a permutation of $$p^{**}(x,n)= \big (x,x,\ldots ,x,1-(\lceil x^{-1} \rceil - 1)x, 0, \ldots , 0 \big )$$, the vector that minimizes Shannon entropy given fixed $$p_1=x$$ (Proposition 2), producing $$H_{\min }(p_1,n)$$.

We then have $$H\big (p'(x,i,n)\big ) \geqslant H\big (p^{**}(x,n)\big )$$, or $$H_{\max }(p_i,n) \geqslant H_{\min }(p_1,n)$$, for *x* in $$(\frac{1}{n},\frac{1}{i})$$. Because $$p'(x,i,n)$$ is not a permutation of $$p^{**}(x,n)$$, the inequality $$H\big (p'(x,i,n)\big ) > H\big (p^{**}(x,n)\big )$$ is strict by the equality condition of Corollary 3.16 of Aw and Rosenberg (2018). $$\square $$

### Proposition B.7

Let $n \geqslant 3$. Consider vectors *p* in $\Delta _{n-1}$ with $p_1 \geqslant p_2 \geqslant \ldots \geqslant p_n$. On the interval $[\frac{1}{n},\frac{1}{n-1}]$, $H_{\max }(p_{n-1},n)=H_{\min }(p_1,n)$.

### Proof

This result is obtained by direct computation of the functions, noting that on the relevant interval, the minimizing vector in Proposition 2 and the maximizing vector in Theorem 3 both have their first $n-1$ components equal to one another. $\square$

### Proposition B.8

Let $n \geqslant 3$. Consider vectors *p* in $\Delta _{n-1}$ with $p_1 \geqslant p_2 \geqslant \ldots \geqslant p_n$. Consider $i_1, i_2$ with $2 \leqslant i_1 < i_2 \leqslant n$. (i) $H_{ \min }(p_{i_1}=0,n)=H_{\min }(p_{i_2}=0,n)=0$. (ii) If $i_1 \geqslant 3$, then $H_{\max }(p_{i_1}=0,n)$ has a positive value that lies strictly below the value of $H_{\max }(p_{i_2}=0,n)$. (iii) If $i_1=2$, then $H_{\max }(p_{i_1}=0,n)=0$.

### Proof

(i) This result is obtained by direct computation.

(ii) If $3 \leqslant i_1 < i_2$, then $H_{\max }(p_{i_1}=0,n)= \log (i_1-1) < \log (i_2-1) = H_{\max }(p_{i_2}=0,n)$.

(iii) If $i_1=2$, then we obtain $H_{\max }(p_{i_1}=0,n)=0$ by direct computation. $\square$

Proposition B.8 describes the overlap of intervals for different $p_i$ along the y-axis, for $2\leqslant i \leqslant n$. In Proposition B.9, we describe the overlap for $0<x \leqslant \frac{1}{i}$. In particular, on the interval $(0,\frac{1}{i}]$, the region between the upper and lower bounds given $p_{i_1}$ overlaps the corresponding region given $p_{i_2}$, where $i_1 < i_2$; in the special case $(i_1,i_2)=(2,n)$, the overlap is a curve.

### Proposition B.9

Let $n \geqslant 3$. Consider vectors *p* in $\Delta _{n-1}$ with $p_1 \geqslant p_2 \geqslant \ldots \geqslant p_n$. Consider $i_1, i_2$ with $2 \leqslant i_1 < i_2 \leqslant n$. Suppose *x* lies in $(0,\frac{1}{i_2}]$. (I) For $(i_1,i_2) \ne (2,n)$, $H_{\max }(p_{i_1}=x,n)> H_{\min }(p_{i_2}=x,n) > H_{\min }(p_{i_1}=x,n)$. (II) For $(i_1,i_2)=(2,n)$, $H_{\max }(p_{2}=x,n) = H_{\min }(p_{n}=x,n)$.

### Proof

We separately consider $\big (0,\frac{1}{n}\big )$, $\frac{1}{n}$, and, $\big (\frac{1}{n},\frac{1}{i_2}\big ]$.

(A) For *x* in $\big (0,\frac{1}{n}\big )$, we claim
  (A.i) $$H_{\max }(p_n=x,n)> H_{\max }(p_{n-1}=x,n)> \ldots > H_{\max }(p_2=x,n)$$,
  (A.ii) $$H_{\max }(p_2=x,n)=H_{\min }(p_n=x,n),$$
  (A.iii) $$H_{\min }(p_n=x,n)> H_{\min }(p_{n-1}=x,n)> \ldots > H_{\min }(p_2=x,n).$$
(B) For $x=\frac{1}{n}$, we claim
  (B.i) $$H_{\max }(p_n=\frac{1}{n},n)=H_{\max }(p_{n-1}=\frac{1}{n},n)=\ldots = H_{\max }(p_2=\frac{1}{n},n)$$,
  (B.ii) $$H_{\max } (p_n = \frac{1}{n},n)=H_{\min }( p_n = \frac{1}{n},n)$$,
  (B.iii) $$H_{\min }(p_n=\frac{1}{n},n)> H_{\min }(p_{n-1}=\frac{1}{n},n)> \ldots > H_{\min }(p_2=\frac{1}{n},n)$$.
(C) For *x* in $\big (\frac{1}{n},\frac{1}{i_2}\big ]$ with $3 \leqslant i_2 \leqslant n-1$, we claim
  (C.i) $$H_{\max }(p_2=x,n)> H_{\max }(p_3=x,n)> \ldots > H_{\max }(p_{i_2}=x,n)$$,
  (C.ii) $$H_{\max }(p_{i_2}=x,n) \geqslant H_{\min }(p_{i_2}=x,n)$$,
  (C.iii) $$H_{\min }(p_{i_2}=x,n)> H_{\min }(p_{i_2-1}=x,n)> \ldots > H_{\min }(p_2=x,n)$$.

(A.ii) proves Proposition B.9.II. The remaining eight statements suffice to prove Proposition B.9.I. For $2 \leqslant i_1 < i_2 \leqslant n$ and $(i_1,i_2) \ne (2,n)$, the inequalities $H_{\max }(p_{i_1}=x,n)> H_{\min }(p_{i_2}=x,n) > H_{\min }(p_{i_1}=x,n)$ are reached by the chain of inequalities specified by statements (A.i), (A.ii), and (A.iii) for *x* in $(0,\frac{1}{n})$, by the chain of inequalities of (B.i), (B.ii), and (B.iii) for $x=\frac{1}{n}$, and by the chain of inequalities of (C.i), (C.ii), and (C.iii) for *x* in $(\frac{1}{n}, \frac{1}{i_2}]$.

(A) Consider *x* in the interval $(0,\frac{1}{n})$.

(A.i) Let $n \geqslant 3$ and suppose $3 \leqslant i \leqslant n$. Using Theorem 3, define a function *f*(*x*, *i*, *n*),

$$\begin{aligned} f(x,i,n) =&H_{\max }(p_i=x,n)-H_{\max }(p_{i-1}=x,n) \\ =&-[1-(n-i+1)x] \log \left[ \frac{1-(n-i+1)x}{i-1}\right] \\&+[1-(n-i+2)x] \log \left[ \frac{1-(n-i+2)x}{i-2}\right] +x \log x. \end{aligned}$$

To prove (A.i), it suffices to prove $f(x,i,n)>0$ for *x* in $(0,\frac{1}{n})$.

To prove $f(x,i,n)>0$, we prove (a) *f* is strictly convex for *x* in $(0,\frac{1}{n})$, or $\frac{\partial ^2}{\partial x^2}f(x,i,n)>0$; (b) *f* has a local minimum at $x=\frac{1}{n}$; (c) $f\big (x=\frac{1}{n},i,n\big )\geqslant 0$. For the continuous function *f*, these three claims suffice to demonstrate $f(x,i,n)>0$ for *x* in $(0,\frac{1}{n})$: if *f* is strictly convex on $(0,\frac{1}{n})$ and has a minimum at $x=\frac{1}{n}$ that is nonnegative, then *f* must decrease monotonically on $(0,\frac{1}{n})$ to a nonnegative value—and hence, $f(x,i,n)>0$ for all *x* in $(0,\frac{1}{n})$.

(a) Taking the second derivative and noting $1-(n-i+2)x \geqslant 1-(n-3+2)\frac{1}{n} = \frac{1}{n} > 0$, we have

$$\begin{aligned} \frac{\partial ^2}{\partial x^2}f(x,i,n) = \frac{1}{x \big [1 - (n-i+1) x\big ] \big [1 - (n-i+2) x\big ]}>0. \end{aligned}$$

(b) By direct computation, $\frac{\partial }{\partial x}f\big (x=\frac{1}{n},i,n\big ) = 0$. Because *f* is strictly convex on $(0,\frac{1}{n})$, this point is a local minimum.

(c) By direct computation, it is straightforward to show that $\lim _{x\rightarrow \frac{1}{n}} f(x,i,n) = 0.$

(A.ii) By direct computation from Theorems 3 and 4,

$$\begin{aligned} H_{\max }(p_2=x,n)=H_{\min }(p_n=x,n){} & {} =-[1-(n-1)x)] \log [1-(n-1)x] \\{} & {} \qquad - (n-1)x \log x. \end{aligned}$$

(A.iii) As we did for (A.i), let $n \geqslant 3$ and suppose $3 \leqslant i \leqslant n$. Using Theorem 4, define *g*(*x*, *i*, *n*),

$$\begin{aligned} g(x,i,n)&= H_{\min }(p_i=x,n)-H_{\min }(p_{i-1}=x,n) \\&= -[1-(i-1)x] \log [1-(i-1)x] +[1-(i-2)x] \log [1-(i-2)x]\\&\quad -x \log x. \end{aligned}$$

To prove (A.iii), it suffices to prove $g(x,i,n)>0$ for *x* in $(0,\frac{1}{n})$.

To prove $g(x,i,n)>0$, we prove (a) *g* is strictly concave for *x* in $(0,\frac{1}{n})$, or $\frac{\partial ^2}{\partial x^2}g(x,i,n)<0$; (b) *g* is non-negative at the boundaries of the interval, $g(0)\geqslant 0$ and $g\big (\frac{1}{n}\big )\geqslant 0$. For the continuous function *g*, these two claims suffice to demonstrate $g(x,i,n) > 0$ for *x* in $(0,\frac{1}{n})$: if *g* is strictly concave on $(0,\frac{1}{n})$, then it has a monotonically decreasing slope, so that if it is non-negative at the start and end of the interval, it cannot decrease to zero on the interior of the interval.

(a) The second derivative, along with $1-(i-2)x> 1-(i-1)x > 1-(n-1)\frac{1}{n} = \frac{1}{n}$, yields

B1 $$\begin{aligned} \frac{\partial ^2}{\partial x^2}g(x,i,n) =\frac{-1}{x \big [1-(i-1)x\big ] \big [1-(i-2)x\big ]}<0. \end{aligned}$$

(b) We show via direct computation that $\lim _{x\rightarrow 0} g(x,i,n) = 0$ and

B2 $$\begin{aligned} \lim _{x\rightarrow \frac{1}{n}} g(x,i,n) =\frac{1}{n}[(n-i+2)\log (n-i+2)-(n-i+1)\log (n-i+1)]. \end{aligned}$$

The function $w(x)=x \log x$ is strictly increasing for $x > 1$; because $n-i+1 \geqslant 1$, $w(n-i+2)-w(n-i+1) > 0$, so that Eq. B2 is positive.

(B) Consider $$x=\frac{1}{n}$$.

(B.i) This statement was proven in Proposition B.5.

(B.ii) This statement was proven in Proposition B.4.

(B.iii) This statement follows from the proof of (A.iii.b) that, for $g(x,i,n) = H_{\min }(p_i=x,n)-H_{\min }(p_{i-1}=x,n)$, $\lim _{x\rightarrow \frac{1}{n}} g(x,i,n) >0$ for $i \geqslant 3$ and $n\geqslant i.$

(C) Consider *x* in the interval $(\frac{1}{n},\frac{1}{i_2}]$, with $3 \leqslant i_2 \leqslant n-1$.

(C.i) Let $n \geqslant 3$ and suppose $2 \leqslant i \leqslant n-2$. Using Theorem 3, define a function $\ell (x,i,n)$

$$\begin{aligned} \ell (x,i,n)&= H_{\max }(p_{i}=x,n)-H_{\max }(p_{i+1}=x,n)\\&= -(1-ix) \log \left( \frac{1-ix}{n-i}\right) +[1-(i+1)x] \log \left[ \frac{1-(i+1)x}{n-i+1}\right] +x \log x. \end{aligned}$$

To prove (C.i), it suffices to prove $\ell (x,i,n) > 0$ for *x* in $(\frac{1}{n},\frac{1}{i+1}]$.

To prove $\ell (x,i,n)>0$, we prove (a) $\ell$ is strictly convex for *x* in $(\frac{1}{n},\frac{1}{i+1})$, or $\frac{\partial ^2}{\partial x^2}\ell (x,i,n)>0$; (b) $\ell$ has a local minimum at $x=\frac{1}{n}$; (c) $f\big (x = \frac{1}{n}, i, n\big ) \geqslant 0$. For the continuous function $\ell$, these three claims suffice to demonstrate $\ell (x,i,n)>0$ for *x* in $(\frac{1}{n},\frac{1}{i+1}]$: if $\ell$ is strictly convex on $(\frac{1}{n},\frac{1}{i+1})$ and has a minimum at $x=\frac{1}{n}$, then $\ell$ must increase monotonically on $(\frac{1}{n},\frac{1}{i+1})$—and hence, $\ell (x,i,n)>0$ for all *x* in $(\frac{1}{n},\frac{1}{i+1}]$.

(a) Taking the second derivative and noting $1-ix$ and $1-(i+1)x$ are positive on $(\frac{1}{n},\frac{1}{i+1})$, we have

$$\begin{aligned} \frac{\partial ^2}{\partial x^2} \ell (x,i,n) = \frac{1}{x(1 - i x) [i -(i+1)x]}>0. \end{aligned}$$

(b) We show by direct computation that $\frac{\partial }{\partial x} f\big (x=\frac{1}{n},i,n\big ) = 0$.

(c) We show by direct computation that $f\big (x=\frac{1}{n},i,n\big )=0$.

(C.ii) $H_{\max }(p_{i_2},n) \geqslant H_{\min }(p_{i_2},n)$ by definition of the bounds in Theorems 3 and 4.

(C.iii) The proof is similar to that of (A.iii), as we continue to use the function *g*(*x*, *i*, *n*) from the proof of that statement. Let $n \geqslant 3$ and suppose $3 \leqslant i \leqslant n-1$. To prove (C.iii), it suffices to prove $g(x,i,n) > 0$ for *x* in $(\frac{1}{n},\frac{1}{i}]$.

To prove $g(x,i,n) > 0$, we prove (a) *g* is strictly concave for *x* in $(\frac{1}{n},\frac{1}{i})$; (b) *g* is positive at the boundaries of the interval, $g(\frac{1}{n}) > 0$ and $g(\frac{1}{i}) > 0$. For the continuous function *g*, these two claims suffice to demonstrate $g(x,i,n) > 0$ for *x* in $(\frac{1}{n},\frac{1}{i}]$: if *g* is strictly concave on $(\frac{1}{n},\frac{1}{i})$, then it has a monotonically decreasing slope, so that if it is positive at the start and end of the interval, it cannot decrease to zero on the interior of the interval.

(a) This statement follows as in (A.iii.a): the second derivative in Eq. B1 is negative on $(\frac{1}{n},\frac{1}{i_2}]$.

(b) This statement follows for the left endpoint $x=\frac{1}{n}$ as in (A.iii.b); for the right endpoint, $x=\frac{1}{i}$, $g(x,i,n) = \frac{2}{i}\log 2 > 0$. $\square$

## References

[CR1] Alcala N, Rosenberg NA (2017). Mathematical constraints on $$F_{ST}$$: biallelic markers in arbitrarily many populations. Genetics.

[CR2] Alcala N, Rosenberg NA (2019). $$G_{ST}$$’, Jost’s $$D$$, and $$F_{ST}$$ are similarly constrained by allele frequencies: a mathematical, simulation, and empirical study. Mol Ecol.

[CR3] Alcala N, Rosenberg NA (2022). Mathematical constraints on $$F_{ST}$$: multiallelic markers in arbitrarily many populations. Philos Trans R Soc B.

[CR4] Aw AJ, Rosenberg NA (2018). Bounding measures of genetic similarity and diversity using majorization. J Math Biol.

[CR5] Beisel J-N, Moreteau J-C (1997). A simple formula for calculating the lower limit of Shannon’s diversity index. Ecol Model.

[CR6] Berry DW, Sanders BC (2003). Bounds on general entropy measures. J Phys A: Math Gen.

[CR7] Chao A, Chiu C-H, Jost L (2014). Unifying species diversity, phylogenetic diversity, functional diversity, and related similarity and differentiation measures through Hill numbers. Annu Rev Ecol Evol Syst.

[CR8] Chao A, Jost L (2015). Estimating diversity and entropy profiles via discovery rates of new species. Methods Ecol Evol.

[CR9] Chao A, Shen T-J (2003). Nonparameteric estimation of Shannon’s index of diversity when there are unseen species in a sample. Environ Ecol Stat.

[CR10] Chave J (2004). Neutral theory and community ecology. Ecol Lett.

[CR11] Cushman SA (2021). Entropy in landscape ecology: a quantitative textual multivariate review. Entropy.

[CR12] Dembo A, Cover TM, Thomas JA (1991). Information theoretic inequalities. IEEE Trans Inf Theory.

[CR13] Garud NR, Rosenberg NA (2015). Enhancing the mathematical properties of new haplotype homozygosity statistics for the detection of selective sweeps. Theor Popul Biol.

[CR14] Hill MO (1973). Diversity and evenness: a unifying notation and its consequences. Ecology.

[CR15] Jakobsson M, Edge MD, Rosenberg NA (2013). The relationship between $$F_{ST}$$ and the frequency of the most frequent allele. Genetics.

[CR16] Jost L (2006). Entropy and diversity. Oikos.

[CR17] Jost L (2010). The relation between evenness and diversity. Diversity.

[CR18] Khan MA, Pečaric D, Pečarić J (2017). Bounds for Shannon and Zipf-Mandelbrot entropies. Mathemat Meth Appl Sci.

[CR19] Leinster T (2021). Entropy and diversity: the axiomatic approach.

[CR20] Leinster T, Cobbold CA (2012). Measuring diversity: the importance of species similarity. Ecology.

[CR21] Liu C, Whittaker RJ, Ma K, Malcolm JR (2007). Unifying and distinguishing diversity ordering methods for comparing communities. Popul Ecol.

[CR22] Magurran AE (2004). Measuring biological diversity.

[CR23] Magurran AE, Henderson PA (2003). Explaining the excess of rare species in natural species abundance distributions. Nature.

[CR24] Marshall AW, Olkin I, Arnold BC (2010). Inequalities: theory of majorization and its applications.

[CR25] Maruki T, Kumar S, Kim Y (2012). Purifying selection modulates the estimates of population differentiation and confounds genome-wide comparisons across single-nucleotide polymorphisms. Mol Biol Evol.

[CR26] Moitinho-Silva L, Nielsen S, Amir A, Gonzalez A, Ackermann GL (2017). The sponge microbiome project. GigaScience.

[CR27] Mosler K (2001). Multidimensional indices and orders of diversity. Commun Ecol.

[CR28] Patil GP, Taillie C (1982). Diversity as a concept and its measurement. J Am Stat Assoc.

[CR29] Pielou EC (1975). Ecological diversity.

[CR30] Rényi A (1961) On measures of entropy and information. In: J. Neyman, editor, Proceedings of the Fourth Berkeley Symposium on Mathematical Statistics and Probability. University of California Press, pp 547–562

[CR31] Rodríguez RA, Herrera AM, Quirós Á, Fernández-Rodríguez MJ, Delgado JD (2016). Exploring the spontaneous contribution of Claude E. Shannon to eco-evolutionary theory. Ecol Modell.

[CR32] Rosindell J, Hubbell SP, Etienne RS (2011). The unified neutral theory of biodiversity and biogeography at age ten. Trends Ecol Evol.

[CR33] Shannon CE (1948). A mathematical theory of communication. Bell Syst Tech J.

[CR34] Sherwin WB, Chao A, Jost L, Smouse PE (2017). Information theory broadens the spectrum of molecular ecology and evolution. Trends Ecol Evol.

[CR35] Sherwin WB, Fornells NPI (2019). The introduction of entropy and information methods to ecology by Ramon Margalef. Entropy.

[CR36] Sherwin WB, Jabot F, Rush R, Rossetto M (2006). Measurement of biological information with applications from genes to landscapes. Mol Ecol.

[CR37] Spellerberg IF, Fedor PJ (2003). A tribute to Claude-Shannon (1916–2001) and a plea for more rigorous use of species richness, species diversity and the ‘Shannon-Wiener’ Index. Glob Ecol Biogeogr.

[CR38] Wong JS, Chan YKS, Ng CS, Tun KP, Darling ES (2018). Comparing patterns of taxonomic, functional and phylogenetic diversity in reef coral communities. Coral Reefs.

