# Supplementary material for: Mathematical bounds on Shannon entropy given the abundance of the ith most abundant taxon
Source: J Math Biol. 2023 Oct 26;87(5):76. doi: 10.1007/s00285-023-01997-3 (PMC10603011; doi:10.1007/s00285-023-01997-3)
Supplement: Supplementary file 1 — (pdf 800 KB) [file 285_2023_1997_MOESM1_ESM.pdf]

Supplemental Materials

Color	Order	Scientific name	Location	H	n	p_1	p_2	p_3	p_{10}	p_{14}	p_{18}
Red	Clionaida	*Cliona delitrix*	USA	1.10	3	0.333	0.333	0.333	0.0000	0.0000	0.0000
Yellow	Dictyoceratida	*Dysidea avara*	Spain	1.11	65	0.488	0.398	0.076	0.0004	0.0003	0.0003
Blue	Haplosclerida	*Amphimedon chloros*	Israel	1.11	1678	0.865	0.010	0.010	0.0032	0.0017	0.00012

Table S1: Three sponge microbiomes highlighted in Figure 6.

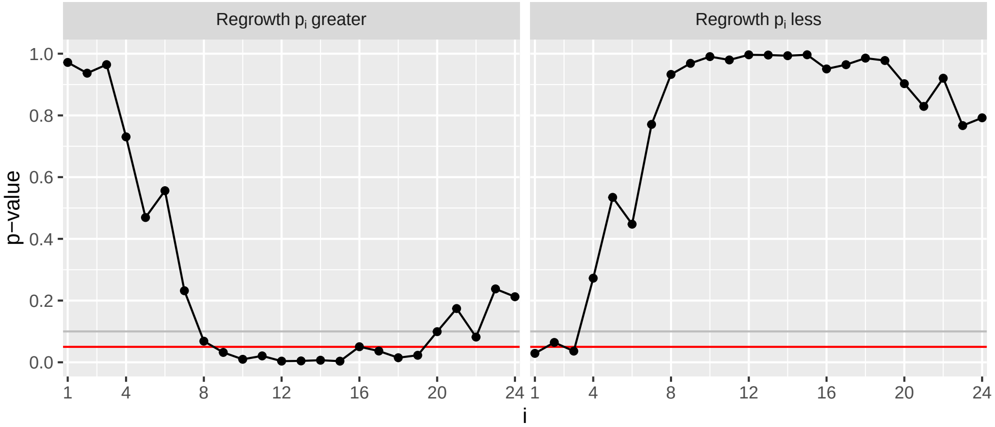

Figure S1: Wilcoxon rank sum P-values for comparisons of p_i between regrowth and non-regrowth reefs, for i from 1 to 24. The red line denotes the $P = 0.05$ significance threshold, and the gray line denotes $P = 0.10$. The left-hand panel presents P-values under the alternative hypothesis that p_i is greater for regrowth reefs than for non-regrowth reefs; the right-hand panel presents P-values under the alternative hypothesis that p_i is lower for regrowth reefs.

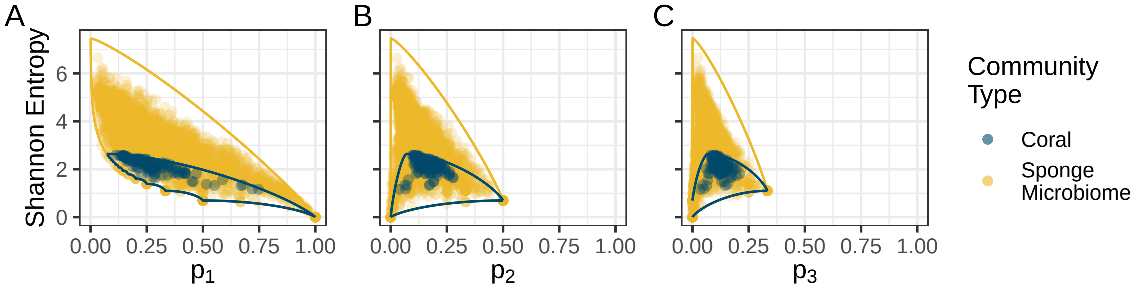

Figure S2: Bounds on Shannon entropy for truncated coral (navy) and sponge microbiome communities (orange), as functions of the abundance of the ith-most abundant taxon. (A) $i = 1$. (B) $i = 2$. (C) $i = 3$. The coral bounds assume $n = 14$, and the sponge bounds assume $n = 1,734$; vectors are truncated to these lengths then normalized to have sum equal to 1.
